# Isolation of *Clostridium limosum* from an outbreak of metritis in farmed mink

**DOI:** 10.1186/s13028-016-0230-7

**Published:** 2016-09-06

**Authors:** Mia Biström, Anna-Maria Moisander-Jylhä, Sirpa Heinikainen, Kirsti Pelkola, Mirja Raunio-Saarnisto

**Affiliations:** 1 Veterinary Bacteriology Research Unit, Finnish Food Safety Authority Evira, Mustialankatu 3, 00790 Helsinki, Finland; 2 Finnish Fur Breeders Association, Outovedentie 129, 69600 Kaustinen, Finland; 3 Veterinary Bacteriology Research Unit, Finnish Food Safety Authority Evira, Neulaniementie 4, 70210 Kuopio, Finland; 4 Production Animal and Wildlife Health Research Unit, Finnish Food Safety Authority Evira, Keskuskatu 23, 60100 Seinäjoki, Finland

**Keywords:** *Clostridium limosum*, Mink, Metritis, Anaerobe infection

## Abstract

**Background:**

An outbreak of sudden death of pregnant farmed mink in Finland occurred during the busiest whelping period in the spring of 2013. The affected farms were all located in western Finland in a rather narrow geographic area, Ostrobothnia. Dead mink from 22 farms were submitted for laboratory diagnostics to the Finnish Food Safety Authority (Evira). The carcasses were necropsied and tissue specimens were prepared for histology. Samples of internal organs and peritoneal fluid were cultured bacteriologically.

**Results:**

Major pathological findings included hemorrhagic vaginal discharge, severely inflamed uteri with luminal hemorrhagic exudate and dead fetuses. Dead fetuses were present in the peritoneal cavity and associated severe peritonitis occurring as sequela of uterine rupture were found in most minks. Histological findings included hemorrhages, neutrophil infiltrations, degenerative inflammatory cells, edema, fibrin and rod-shaped bacteria on all layers of the uterine wall. In most samples abundant and pure anaerobic bacterial growth of *Clostridium limosum* was found.

**Conclusions:**

This is the first report of *C. limosum* associated metritis in farmed mink. Disease was only observed in pregnant females and the uterus was the primary site of infection. The source of infection and the route of transmission remained unclear, but feed borne transmission was suspected.

## Findings

Clostridia are anaerobic rod-shaped bacteria with a world-wide distribution. Several clostridial species have been found in soil, freshwater and marine sediments. Some of these are able to cause endogenous infection from wound contamination or by ingestion. Several clostridial species are also part of the normal flora of the lower intestinal tract of both animals and man [1]. *Clostridium limosum* is a soil bacterium that is able to degrade animal tissue and cause gas gangrene [2]. It has been reported in a few cases of severe infections in both animals and man. In most cases, *C. limosum* was isolated as a part of a mixed infection [2–6].

Here we report an outbreak of metritis in farmed mink in Finland. The outbreak took place during the busiest whelping period in the end of April 2013. Several mink farmers and their veterinarians contacted the diagnostic laboratory because of a sudden increased mortality of pregnant female mink. All the affected farms were located in western Finland in a rather small geographic area, Ostrobothnia. One to three carcasses from 22 farms were submitted for diagnostic examination. In many cases the female minks were reported to have had bloody vaginal discharge but several minks were also found dead without previous signs of illnesses. No other minks than the pregnant females were reported to be affected. The morbidity and mortality in one of the affected farms reached 10 and 5 %, respectively. All the affected farms had the feed delivered from two feeding kitchens having a common source of feedstuff. Approximately half of the affected farms had Aleutian disease free status and all the farms vaccinate against botulism. The affected mink were of different pelt colors and in normal body condition. Unusually wet or cold weather, which could have predisposed the mink to the disease, did not occur.

The mink sent for examination were pregnant and close to whelping date. All mink had bloody vaginal discharge. The uterine wall was thin, moist and fragile and the uterine lumen was filled with a hemorrhagic exudate. Both uterine horns were affected. Several fetuses were present in most uteri. The fetuses varied in crown-rump length (CRL) from 3 to 7 cm compared to the normal CRL of newborn mink fetuses of approximately 7 cm. Some of the fetuses were macerated. The uterus was ruptured in one or more locations in most mink leading to the presence of fetuses in the abdominal cavity, peritoneal hemorrhagic exudate and a widespread fibrinous peritonitis. In cases with a non-ruptured uterus, pathological changes did not occur outside the uterus. Lesions in non-abdominal tissues were not observed in any case. Thus the uterus seemed to be the primary site of infection and other organs were only secondarily affected after uterine rupture. Additionally, field necropsies were performed by veterinarians, who reported similar findings.

**Fig. 1:**
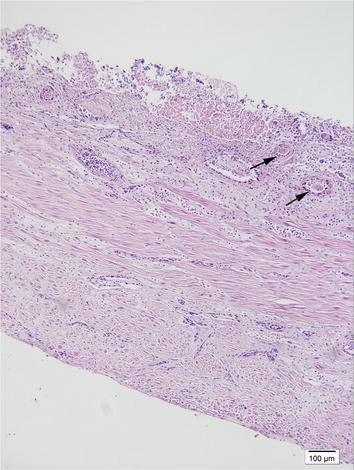
Photomicrograph showing uterine wall in a mink. Uterine lumen at the* top*, serosal surface of the uterus at the* bottom*. Disseminated areas of inflammation on the myometrium and fibrin thrombosis in the vessels (*arrows*) can be seen. The endometrium is mainly degraded and there is necrotic cell debris in the uterine lumen. Hematoxylin and eosin, ×100 magnification

**Fig. 2:**
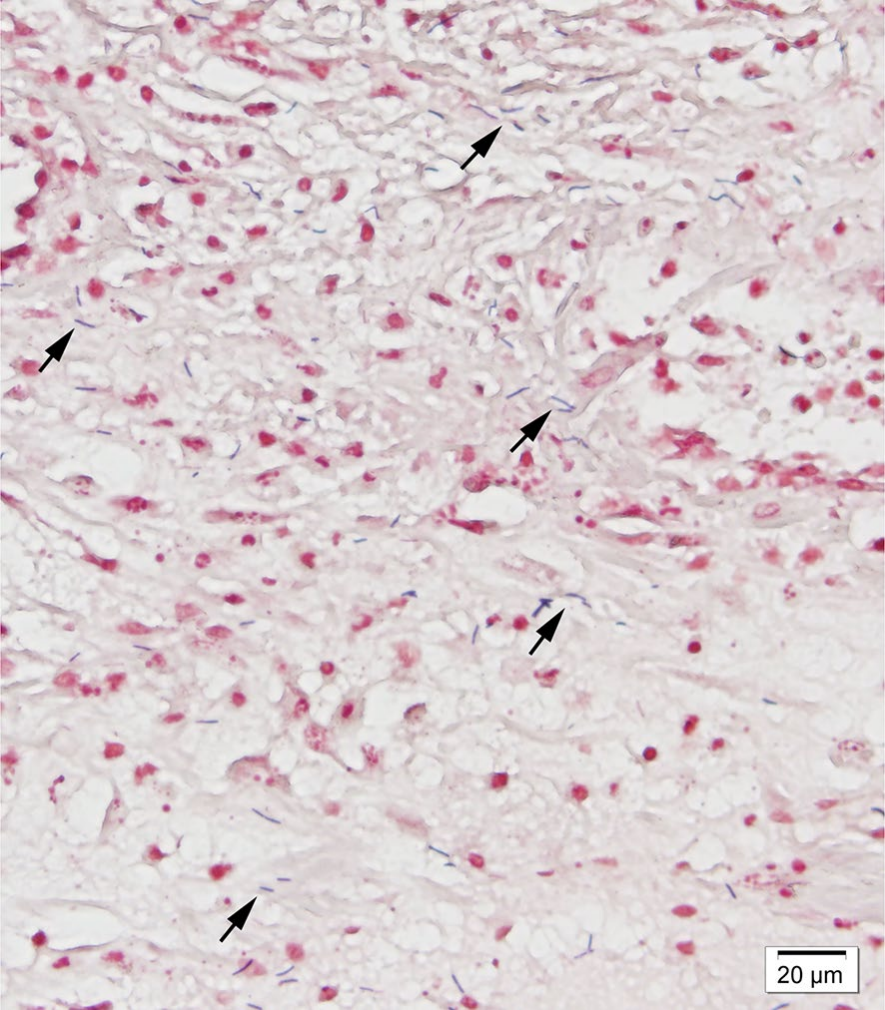
Photomicrograph showing uterine wall in a mink. Detail from Fig. 1. Numerous Gram-positive rod shaped bacteria (*arrows*) are present in all inflamed layers of the uterus. Gram-stain, ×400 magnification

Specimens of uterus, liver, spleen, kidneys, lungs and heart were prepared for histological examination according to routine procedures and stained with hematoxylin and eosin. Metritis being characterized by presence of rod-shaped bacteria, neutrophil infiltration, degenerative inflammatory cells, fibrin and hemorrhages in the endometrium and myometrium were present in all cases (Figs. 1, 2). In some mink with uterine rupture, foci of suppurative inflammation and presence of rod-shaped bacteria were observed only on the serosal surface of the uterus and other abdominal organs. In some cases of severe peritonitis, necrotizing hepatitis was found as well.

Samples of uterus, liver, spleen, kidneys, lungs, heart and peritoneal fluid were cultured on bovine blood agar plates and incubated for 2–4 days in 37 °C both aerobically and with 5 % CO_2_-atmosphere. The plates were examined daily. The samples were also cultured on fastidious anaerobe agar (FAA) plates that were incubated in an anaerobic atmosphere for 4–7 days at 37 °C. The FAA plates were read every second day. Finally, the samples were examined for *Salmonella* by the MSRV method [7–9] and for *Campylobacter* by culturing on CCD agar (Campylobacter blood-free selective medium) in a microaerophilic atmosphere at 37 °C for 4–7 days.

In samples from mink from 17 of the farms, abundant and pure anaerobic bacterial growth in the uterus and peritoneal fluid was observed. No significant aerobic bacterial growth was detected. The bacteria grew in anaerobic atmosphere on FAA agar as a spreading, hemolytic, catalase-negative growth. The bacteria stained Gram-positive and were morphologically large, straight rods. A few subterminal spores were observed. API20A (Biomérieux, Marcy-l’Etoile, France) analyses gave profile 00020003: *Clostridium histolyticum/Clostridium* sp. 16S rDNA was amplified and sequenced with primers 8FX and 1407R [10] thus revealing the bacterium as *C. limosum.* The sequenced strain was later also identified as *C. limosum* by MALDI-TOF MS (Bruker Daltonics GmbH MALDI Biotyper 3.1) with a species level score of 2.198.

This is the first report of *C. limosum* isolated from metritis in farmed mink. An underlying cause was not found. Previously, *C. limosum* has been reported only in a few opportunistic infections and mostly as part of a mixed infection. Our findings indicate that *C. limosum* may also cause outbreaks affecting many individuals and at several locations simultaneously. Since no other pathogens were found consistently, it is likely that *C. limosum* was responsible for the outbreak.

In the previous year (2012), a disease outbreak with similar symptoms occurred in Finnish mink farms, but at that time the causative agent was not identified. Most of the affected farms were the same as in the outbreak of 2013. Miscellaneous aerobic bacterial infections and in some cases also abundant growth of *Clostridium* sp. was detected in the 2012 outbreak. A bacterium stored from the 2012 outbreak was reexamined by MALDI-TOF MS after the 2013 outbreak and was identified as *C. limosum* with a species level score of 2.158.

An outbreak of necrotizing metritis was reported in Danish mink in 2000 [11]. The causative agent was shown to be *Salmonella* Dublin. Also *Campylobacter jejuni* has been reported to cause abortions in mink, but no severe metritis and deaths of the females as in our case was observed [12].

The source of infection and the route of transmission remain unclear. A food borne origin was strongly suspected, but could not be confirmed. The feedstuff was not analyzed in a way that allowed separation of *C. limosum* from other anaerobic sulfite reducing bacteria, mainly other clostridia. The raw material of feed contains offal from poultry, pork and beef industries, including alimentary tracts that was treated with formic acid and freezed before being used for feeding. Since *C. limosum* can be found in soil and the lower alimentary tract of several animal species and the treatment with formic acid and freezing is not sufficient to inactivate it, its presence in the feedstuff is possible. Transmission directly from soil or water is not likely since the affected farms were located several kilometers apart and each farm has individual water supply. The females were mated with the males of the same farm, and animals from separate farms had not been in contact, which rules out venereal transmission.

Antimicrobial treatment with trimethoprim sulfadiazine (Oriprim™ trimethoprim 20 mg/g and sulfadiazine 100 mg/g, 3 kg per 1000 kg of feed) was given to all animals 1 day after the first females were found dead and before the causative agent was isolated. After this no new cases were reported.

In this outbreak, disease was only observed in the pregnant females. The uterus seems to be the primary site of infection since other peritoneal organs only had lesions in case of uterine rupture. Pregnancy was the only known factor predisposing animals to disease. *Clostridium limosum* should be considered as a possible cause of metritis in mink.
